# Supplementary material for: Content-rich biological network constructed by mining PubMed abstracts
Source: BMC Bioinformatics. 2004 Oct 8;5:147. doi: 10.1186/1471-2105-5-147 (PMC528731; doi:10.1186/1471-2105-5-147)
Supplement: Additional File 2 — The original results of the above study (non-essential files are deleted to keep the file size under the limit set by BMC bioinformatics). [file 1471-2105-5-147-S2.bz2 › chilibotAdditionalFile2/dip20/22ID9129204E80/html/FADD_TNFRSF1A.html]

 


 **FADD** and **TNFRSF1A** 
  
Found 377 abstracts in PubMed, retrieved 20.  
 

 What does Google say? 
 PDF only 
| .edu only 

---

**Interactive relationship** (e.g. stimulation, inhibition, etc)

**Stimulatory relationship**- In addition, NF kappaB downstream of TNFR  [ **TNFRSF1A** ]  was required for upregulation of Fas  [ **FADD** ]  expression by endogenous TNF secreted in response to TCR stimulation.  Ref: 12813034 J Biol Chem< MedlineTA>J Biol Chem,
- Expression of TNFR1  [ **TNFRSF1A** ]  gene increased 2.7 fold and expression of Fas  [ **FADD** ]  gene decreased 3.1 fold compared to intact endosalpinx, which indicates induction of apoptosis triggered by tumor necrosis factor alpha and inhibition of the Fas  [ **FADD** ]  dependent pathway.  Ref: 12802421 Bull Exp Biol Med< MedlineTA>Bull Exp Biol Med,
**Neutral relationship**- Apoptosis induced by TNF receptor I TNFR1  [ **TNFRSF1A** ]  is thought to proceed via recruitment of the adaptor  **FADD**  and caspase 8 to the receptor complex.  Ref: 12887920 Cell, 2003
- Fas  [ **FADD** ]  CD95 or APO 1 is a member of tumor necrosis factor receptor TNFR  [ **TNFRSF1A** ]  family.  Ref: 12806430 Drug News Perspect, 2001
- The Fas  [ **FADD** ]  associated death domain  **FADD**  adaptor protein  **FADD**  Mort 1  [ **FADD** ]  is recruited by several members of the tumor necrosis factor receptor TNFR  [ **TNFRSF1A** ]  superfamily during cell death activated via death receptors.  Ref: 12815462 Cell Death Differ, 2003
- Neutralizing anti TNF R1  [ **TNFRSF1A** ]  antibodies inhibited macrophage activation assessed by Fas  [ **FADD** ]  L expression and NO secretion.  Ref: 12869351 Arterioscler Thromb Vasc BiolArterioscler Thromb Vasc Biol, 2003

**Non-interactive relationship** (e.g. studied together, co-existance, homology, etc.)

- cFLIP down regulation induced by cFLIP antisense oligodeoxynucleotides sensitized HLE cells to Fas  [ **FADD** ] , TNF R  [ **TNFRSF1A** ] , and TRAIL R mediated apoptosis.  Ref: 12861043 Lab Invest, 2003
- Significantly increased apoptosis of PHA stimulated T cells was observed in COPD annexin positive 75.0 14.7% SD vs. control 50.2 21.8% SD, P = 0.006, along with upregulation of TNF alpha TNFR1  [ **TNFRSF1A** ] , Fas  [ **FADD** ] , and TGFR.  Ref: 12851215 Am J Physiol Lung Cell Mol Physiol< MedlineTA>Am J Physiol Lung Cell Mol Physiol, 2003
- Fas  [ **FADD** ]  CD95, APO 1 is regarded as the prototypical cell death receptor of the TNFR  [ **TNFRSF1A** ]  superfamily.  Ref: 12794152 J Immunol, 2003
- We tested the hypothesis that activation of specific apoptotic pathways represents a mechanism for tubular cell death after CAD kidney transplantation.METHODS Serial tissue sections from paraffin embedded needle biopsy specimens obtained at approximately 1 hr of reperfusion after transplantation of 13 CAD and 12 living related donor LRD renal allografts were examined by using the terminal deoxynucleotide transferase mediated dUTP nick end labeling assay to detect apoptosis and by immunohistochemistry for expression of key pro apoptotic molecules Bax, Bak, tumor necrosis factor receptor TNFR 1  [ **TNFRSF1A** ] , Fas  [ **FADD** ] , and cytochrome c.  Ref: 12865785 Transplantation, 2003
- The chicken death receptors, Fas  [ **FADD** ] , TNFR1  [ **TNFRSF1A** ] , DR6, and TVB, are constitutively expressed in a relatively wide variety of hen tissues.  Ref: 12878204 Biochem Biophys Res Commun, 2003
- Because cellular FLICE caspase 8 inhibitory protein cFLIP is a recently identified intracellular inhibitor of caspase 8 activation that potently inhibits death signaling mediated by all known death receptors, including Fas  [ **FADD** ] , TNF receptor TNF R  [ **TNFRSF1A** ] , and TNF related apoptosis inducing ligand receptors TRAIL Rs, we investigated the expression and function of cFLIP in human HCCs.  Ref: 12861043 Lab Invest, 2003
- While recruitment of death domain DD containing adaptors such as Fas  [ **FADD** ]  associated death domain  **FADD**  and TNFR  [ **TNFRSF1A** ]  associated DD TRADD can lead to the activation of a signal transduction pathway that induces apoptosis, recruitment of TRAF family proteins can lead to the activation of transcription factors such as, NF kappaB and JNK thereby promoting cell survival and differentiation as well as immune and inflammatory responses.  Ref: 12787559 Cytokine Growth Factor Rev, 2003
- CONCLUSIONS IL 6 in contrast to its anti apoptotic modulation of the Fas  [ **FADD** ]  induced pathway exerted a pro apoptotic effect on the TNFalpha actinomycin D induced apoptosis by increasing the number of TNF R  [ **TNFRSF1A** ]  on hepatocytes.  Ref: 12763364 J Hepatol, 2003
- By contrast, in neurons, reovirus induced apoptosis involves a wider array of DRs, including TNFR  [ **TNFRSF1A** ]  and Fas  [ **FADD** ] , and the mitochondria appear to play a less critical role.  Ref: 12766474 Apoptosis, 2003
- As several pathways are involved in induction of apoptosis of T cells, including transforming growth factor TGF beta TGF receptor TGFR, TNF alpha TNFR1  [ **TNFRSF1A** ] , and Fas  [ **FADD** ]  Fas  [ **FADD** ]  ligand, these mediators were also investigated in peripheral blood samples from these subject groups.  Ref: 12851215 Am J Physiol Lung Cell Mol Physiol< MedlineTA>Am J Physiol Lung Cell Mol Physiol, 2003
- Thus, p75 NTR may function as a death receptor similar to Fas  [ **FADD** ]  or TNFR  [ **TNFRSF1A** ]  I or a dependence receptor similar to deleted in colorectal cancer DCC or uncoordinated gene 5 homologues 1 3 UNC5H1 3.  Ref: 12787561 Cytokine Growth Factor Rev, 2003
- Noggin overexpression leads to reduction of apoptosis and retardation of cell differentiation in the eyelid epithelium, which is associated with downregulation of expression of the apoptotic receptors Fas  [ **FADD** ] , p55 kDa TNFR  [ **TNFRSF1A** ] , Id3 protein and keratinocyte differentiation markers loricrin, involucrin.  Ref: 12805214 EMBO J< MedlineTA>EMBO J, 2003
- Based on the structural and functional differences between p75NTR and the related receptors Fas  [ **FADD** ]  or TNFR  [ **TNFRSF1A** ] , it has been suggested that these receptors have distinct signaling functions.  Ref: 12852261 Vitam Horm, 2003
- on the other hand, its death domain is of type II unlike that of Fas  [ **FADD** ]  or TNFR  [ **TNFRSF1A** ]  I, and it has also been shown to be capable of mediating cell death in response to the withdrawal of ligand.  Ref: 12787561 Cytokine Growth Factor Rev, 2003
- The fusion of the transmembrane domain of LMP1 to signaling domains of CD40, TNF R1  [ **TNFRSF1A** ]  and Fas  [ **FADD** ]  activates their signaling.  Ref: 12805217 EMBO J, 2003
